# Surgery Taught by the Cinematograph

**Published:** 1899-12

**Authors:** 


### Surgery Taught by the Cinematograph.

The kinetoscope, which is almost as well known by its French name, cinematograph, and by various trade names, such as the biograph, the vitascope, etc., has already justified its existence as a means of amusement; but it is probable that it has a more useful future than that of mere entertainment. Photography itself, now the adjunct of so many scientific processes, passed at first through very much the same stage. The kinetoscope will doubtless be largely used in the teaching of technical processes. It is already about to be so used in surgical instruction in Paris, and Dr. Doyen, who claims to have suggested and originated this use, tells in *La Science Francaise* (Paris, October 27), of some of the benefits that this profession, as well as the public at large, is to reap from the plan. Dr. Doyen says that several years ago he was desirous of applying cinematography to the teaching of surgery, but the obstacles were then insurmountable. These obstacles no longer exist, and moving pictures of an actual operation performed indoors may now be obtained. Books can not take the place of such pictures. The most detailed descriptions, accompanied by diagrams and photographs, are insufficient. But if a typical operation be photographed with the cinematograph, it may be made clear in less than a minute to a thousand people, where otherwise a whole lecture would be needed to explain it to a small number of students seated close to the professor. The doctor continues:

"Medical literature has been loaded, little by little, with useless discussions and insufficient descriptions that make it impossible for us to appreciate new methods at their proper value. Even surgeons who are able to travel and visit the principal centers of learning, can not always profit by their experience as they might desire to do.

"The unfavorable conditions in which persons who witness a great operation are situated, do not enable more than fifteen or twenty of them to follow with profit the technical details that are of chief interest to them.

"It is necessary for the security of the patient to place the spectators at a distance of at least two meters—six and one-half feet; the hands of the surgeon and his assistants hide a part of the field of view, and the most delicate manœuvers can be seen only by the operator himself.

"Finally, it is not sufficient, if we wish to understand an operatory process, to see the operation performed by a surgeon who has studied under the originator of the process; we must be present at one or several operations performed by the practitioner who has devised the technical details; in a word, we must see the master himself. The surgeon is judged by his work, and the best illustrated publications can not reproduce the personality of the operator, which is his most important quality.

"It is with the aim of filling this regrettable need in surgical instruction that I have studied the question of cinematographical reproduction."

The first demonstration of the teaching of surgery with the cinematograph was made before the members of the British Medical Association at their Edinburgh meeting, July, 1898. This demonstration, says Dr. Doyen, was conclusive, and the new method met with the approval of all the physicians present. Since this first demonstration he has made many others successfully at other scientific gatherings. There has just been announced, moreover, the establishment, under the Paris Faculty of Medicine, of a course in technical operative surgery with cinematographical illustrations. This course is not under Dr. Doyen's superintendence, but is to be managed by other surgeons who claim priority in the application of his idea. This claim he indignantly repudiates, and the customary contest will doubtless enliven French medical circles for some time to come. At any rate, the method seems to have come to stay. Some of the benefits that will result from its adoption, according to the writer, are the ease with which students in far-off countries can familiarize themselves with the practice of the masters of surgical science; the possibility of preserving the films indefinitely as records, forming a pictorial history of surgery; the information that surgeons can derive regarding their own operations, enabling them to correct errors and improve methods; and the possibility of giving the interested public, whom it would be injudicious to admit to the operation itself, general ideas on the subject of surgical procedure.—*Literary Digest*, November 25, 1899.

